# ApoB and LDL partially explain the association between family history of diabetes and lower clinical pregnancy in women who conceived with PCOS

**DOI:** 10.3389/fnut.2026.1819555

**Published:** 2026-05-25

**Authors:** Hang Ge, Hui Chang, Zhao-Xuan Sun, Mu-Xin Guan, Jian-Nan Yu, An-Qi Li, Meng-Yi Zhu, Jing-Shu Gao, Xiao-Ke Wu

**Affiliations:** 1 The First Affiliated Hospital of Zhejiang Chinese Medical University (Zhejiang Provincial Hospital of Chinese Medicine), Hangzhou, China; 2 Zhejiang Chinese Medical University, Hangzhou, China; 3 Department of Obstetrics and Gynaecology, First Affiliated Hospital, Heilongjiang University of Chinese Medicine, Harbin, China; 4 Harbin Institute of Technology, Harbin, China; 5 Heilongjiang University of Chinese Medicine, Harbin, China

**Keywords:** ApoB, clinical pregnancy, family history of diabetes, LDL, lipid metabolism, PCOS, predictive mediation analysis

## Abstract

**Purpose:**

To investigate whether family history of diabetes (FHD) is associated with reproductive outcomes in women with polycystic ovary syndrome (PCOS), and to explore whether baseline metabolic biomarkers statistically explain part of this association.

**Materials and methods:**

We performed a secondary analysis of the PCOSAct trial. Outcomes were first summarized in the full trial cohort, and the primary post-conception analysis was then restricted to the 320 women who conceived. Modified Poisson regression with robust standard errors was used to estimate risk ratios (RRs) for the association between FHD and clinical pregnancy. E-values were calculated to assess the potential influence of unmeasured confounding. Predictive mediation analyses were performed to evaluate whether baseline lipid parameters statistically explained part of this association. Restricted cubic splines (RCS) were used to examine the dose–response relationships.

**Results:**

Among women who conceived, those with a positive FHD had a lower clinical pregnancy rate than those without FHD (57.8% vs. 70.7%, *p* = 0.048), whereas conception rates were similar in the full cohort. Live birth was also numerically lower in the FHD-positive group among women who conceived, but this difference did not reach statistical significance. Positive FHD was also associated with a less favorable metabolic features, including higher body mass index, waist-to-height ratio, LDL, triglycerides, and ApoB, and lower HDL (all *p* < 0.05). In modified Poisson models, the association between positive FHD and clinical pregnancy was directionally negative (Model 1: RR = 0.82, 95% CI: 0.65–1.02; Model 5: RR = 0.85, 95% CI: 0.66–1.08). Predictive mediation analyses revealed that LDL and ApoB statistically explained part of this association, accounting for 21.1 and 25.3%, respectively. RCS analyses further showed inverse dose-dependent associations of ApoB and LDL with clinical pregnancy.

**Conclusion:**

A positive FHD was associated with lower clinical pregnancy rates among women with PCOS who conceived. This association might be partly explained by elevated ApoB and LDL levels, although the modified Poisson models and E-value analyses support a cautious interpretation. These findings suggest that elevated ApoB and LDL are associated with poorer post-conception reproductive outcomes and may statistically explain part of the observed association.

## Introduction

Polycystic ovary syndrome (PCOS) is one of the most common endocrine disorders (1) in women of reproductive age, affecting 6–10% worldwide (2). It is characterized by oligo-ovulation, clinical and/or biochemical hyperandrogenism (HA), and polycystic ovarian morphology, which usually lead to infertility. PCOS is often complicated by metabolic disturbances. Obesity is highly prevalent among women with PCOS, who also exhibit a high rate of insulin resistance (IR) (26.7%) four-fold increased risk of developing Type 2 diabetes mellitus (T2DM) before the age of 40 (3). Although the etiology of PCOS remains unclear, current evidence suggests it is a polygenic disorder with epigenetic, developmental, and environmental components (4).

Women with PCOS usually face a heightened risk of adverse reproductive outcomes, including anovulation-related infertility, abortion, pregnancy difficulties, and preterm birth (5–7).

Growing evidence indicates that even after achieving a positive pregnancy test [detectable serum human Chorionic Gonadotropin (hCG)], PCOS predisposes the pregnant women (or patients) to several pregnancy complications, such as pregnancy loss (8, 9) and gestational diabetes (10). These may be caused by high body mass index (BMI) and IR in PCOS patients (10–12). For instance, in pregnant rats with PCOS-like features, a cluster of mitochondria-ROS-SOD1/ Nrf2 alterations in the placenta was linked to harmful effects of IR on embryo survival (13). Consistently, elevated fasting insulin and IR are more frequently observed in women with spontaneous recurrent pregnancy loss (14). Thus, the impaired insulin signaling and lipid abnormalities likely contribute to poor reproductive outcomes in PCOS populations.

A positive family history of diabetes (FHD) is a potential risk factor for metabolic dysfunction and PCOS. Previous studies have reported the prevalence of FHD in PCOS, accounting for over one-third of the population (15). Current evidence suggests that T2DM can independently influence the metabolism of offspring (15–17), including central fat accumulation, prediabetes, metabolic syndrome (MS), impaired fasting glucose, and creatinine, etc. FHD in first-degree relatives would also lead to gestational diabetes mellitus (GDM) with significantly elevated levels of BMI, HbA1c and random plasma glucose (18). Notably, these metabolic abnormalities commonly found in PCOS are also observed in their female and male first-degree relatives (4). A recent study (19) further demonstrated that existence of diabetes in parents and grandmother would increase the risk of GDM in women without chronic disease, and diabetes in father was a risk factor of GDM even in lean pregnant women. Collectively, FHD has a significant effect on metabolic disturbances of PCOS, which needs further investigation into its impact on reproductive outcomes. Notably, many of these metabolic features, particularly lipid abnormalities, are modifiable through nutritional and lifestyle interventions, underscoring the need to understand their role as potential biomarkers linking non-modifiable genetic risk to reproductive outcomes.

Dyslipidemia is another common feature in both PCOS (20–22) and women with FHD (23), and circulating lipid profiles, including LDL and apolipoprotein B (ApoB), are key modifiable biomarkers influenced by diet and lifestyle. Among women with PCOS, dyslipidemia is negatively associated with cumulative live birth rate (CLBR) after adjustment for confounders (24). Specifically, total cholesterol (TC) has been shown to adversely affect CLBR following the first ovarian stimulation in PCOS (25). Overweight and higher serum TC have also been identified as risk factors for poor outcomes in *in vitro* Fertilization/Intracytoplasmic Sperm Injection (IVF/ICSI) cycles among PCOS (26). In couples planning IVF, elevated TC or low-density lipoprotein (LDL) in both partners were associated with educed live birth rates compared to those with normal lipid profiles (27). In contrast, high-density lipoprotein (HDL) appears to be beneficial for clinical pregnancy (28). Therefore, we hypothesize that dyslipidemia may act as key factors in the pathway between FHD and reproductive outcomes in PCOS.

In summary, previous studies have demonstrated that FHD contributes to metabolic abnormalities in women, and women with PCOS frequently exhibit endocrine and metabolic disorders, which are often negatively correlated with fertility outcomes. However, limited evidence exists regarding how FHD affects reproductive outcomes in PCOS through modifiable metabolic profiles. Adopting an integrated perspective that links family history and endocrine risk to reproductive health via circulating metabolic biomarkers is essential for identifying potential targets for preconception intervention. Therefore, the aim of this study was to evaluate the relationship between FHD and reproductive outcomes in women with PCOS, and to explore whether baseline lipid profiles may serve as intermediate metabolic biomarkers associated with this relationship. Identifying such biomarkers may help improve risk stratification before conception in this high-risk population.

## Materials and methods

### Participants

This study was a secondary analysis of the Acupuncture and Clomiphene in Polycystic Ovary Syndrome Trial (PCOSAct), a randomized clinical trial conducted in mainland China. PCOSAct enrolled 1,000 patients who were diagnosed with PCOS using the modified Rotterdam criteria (29, 30): subjects with oligomenorrhea (menstrual interval ≥35 days and/or ≤8 menses 1 year) or amenorrhea (more than 90 days between two menstrual cycles) combined with clinical hyperandrogenism (modified Ferriman-Gallwey score ≥5) and/or polycystic ovaries (>12 antral follicles (≤9 mm) and/or ovarian volume >10 mL by ultrasound). The participants were randomly assigned to one of four groups, each 250, and received acupuncture/sham acupuncture plus clomiphene/placebo for a duration of 4 menstrual cycles. The primary findings, inclusion and exclusion criteria, and detailed study design have been published (31, 32). As two individuals dropped out during the intervention, a total of 998 patients and their clinical data were analyzed. This trial was registered at ClinicalTrials.gov (No. NCT01573858, Date: 2012-07-06), and approved by the Regional Ethics Committee of the First Affiliated Hospital of Heilongjiang University of Traditional Chinese Medicine, Harbin, China (No. 2010HZYLL-010). Both participants and their partners provided written informed consent before participating in the study.

### Clinical measurement

All biochemical assays were conducted in the main laboratory. The assays and anthropometric measurement at baseline were performed after an overnight fast. All hormone assays utilized fasting blood samples collected at baseline. Key baseline metabolic parameters, including lipids and lipoproteins, were assessed: HDL, LDL, triglyceride, apolipoprotein A1 (ApoA1), ApoB, TC, and lipoprotein (a). Clinical indices were calculated as follows: BMI = weight (kg)/height^2^ (m^2^); waist to hip ratio (WHR) = waist measurement (cm)/hip measurement (cm); waist to height ratio (WHtR) = waist measurement (cm)/height (cm); free androgen index (FAI) = total testosterone (nmol/L) × 100/ sex hormone binding globulin (SHBG, nmol/L); luteinizing hormone (LH) to follicle-stimulation hormone (FSH) ratio (LFR) = LH (mIU/mL)/FSH (mIU/mL) ratio; homeostasis model assessment of insulin resistance (HOMA-IR) = fasting glucose (mmol/L) × fasting insulin (μU/mL)/22.5.

MS was diagnosed according to Chinese guidelines (33), when three or more of the following criteria were met: (1) central obesity: waist circumference ≥85 cm in women; (2) hyperglycemia: fasting plasma glucose ≥6.1 mmol/L or 2-h postload glucose ≥7.8 mmol/L and/or previous diagnosis of diabetes; (3) hypertension: blood pressure ≥130/85 mmHg and/or previously diagnosed hypertension; (4) fasting triglycerides ≥1.70 mmol/L; (5) fasting HDL < 1.04 mmol/L. Polycystic ovary morphology (PCOM) was diagnosed when there were 12 or more follicles measuring 2–9 mm in diameter at least one ovary (34). HA included clinical HA and biochemical HA; clinical HA was defined as modified Ferriman–Gallwey score ≥5 (30), and biochemical HA was defined as total testosterone ≥1.67 nmoL/L or an FAI > 5 (35).

Ovulation was defined as any elevated progesterone level (>3 ng/mL) per cycle (36). Conception was defined as a positive serum hCG test. Clinical pregnancy was defined as intrauterine pregnancy with fetal heart pulsation detected by transvaginal ultrasonography. Live birth was defined as delivery of a viable infant.

### Statistical analysis

**Figure 1:**
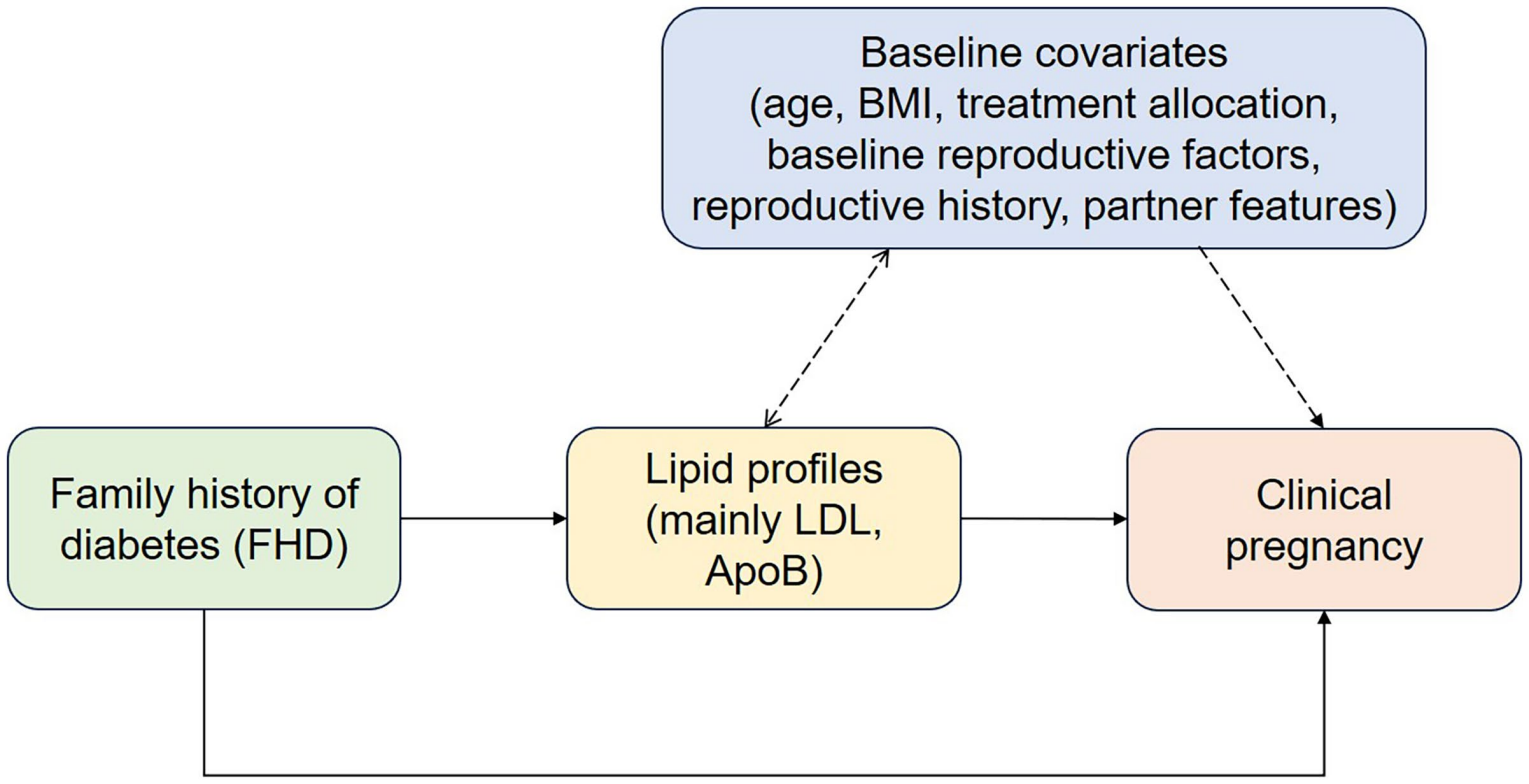
Simplified directed acyclic graph showing the assumed relationships among family history of FHD, lipid parameters, baseline covariates, and clinical pregnancy in women with PCOS who conceived.

Categorical variables are presented as frequencies and percentages, and continuous variables are presented as means and standard deviations. Between-group comparisons were performed using the Kruskal-Wallis rank sum test or Pearson chi-square test, as appropriate. Covariates were selected *a priori* based on clinical relevance, previous literature, and a directed acyclic graph (DAG) representing the assumed relationships among family history of diabetes, lipid parameters, baseline covariates, and clinical pregnancy (Figure 1). Modified Poisson regression with robust standard errors was used to estimate risk ratios (RRs) and 95% confidence intervals (CIs) for the association between FHD and clinical pregnancy among women who conceived. E-values were calculated for the modified Poisson estimates to assess the minimum strength of association that an unmeasured confounder would need to have with both FHD and clinical pregnancy to fully explain the observed association. Predictive mediation analyses were conducted to assess whether baseline lipid parameters (LDL, ApoB, HDL, and triglyceride) statistically explained part of the association between FHD and clinical pregnancy. Bootstrap with 1,000 resamples was used. Restricted cubic spline (RCS) models were used to evaluate dose–response and potential non-linear associations of ApoB and LDL with clinical pregnancy. Statistical significance was set at *p* < 0.05. All statistical analysis was performed using R 4.5.1 and SPSS 26.0.

## Results

This study enrolled 1,000 women with PCOS, with 320 women conceived. The participants were divided into two groups based on their FHD. Table 1 presents baseline characteristics of conceived participants stratified by FHD status. There was no significant difference in age, height, blood pressure, glucose and insulin metabolism. Individuals with positive FHD exhibited significantly higher body weight, waist and hip circumference, BMI and WHtR. Key lipid metabolic parameters were significantly altered in the FHD-positive group, characterized by higher levels of LDL, triglyceride, and ApoB, as well as lower HDL (all *p* < 0.05). The patients in FHD-positive group exhibited lower progesterone and SHBG levels, and younger age at menarche (all *p* < 0.05). Additionally, they showed a higher proportion of amenorrhea (17.2% vs. 6.3%, *p* = 0.010), while other clinical phenotypes (MS, hypertension, hyperandrogenism, and PCOM) revealed no statistical difference.

**Table 1:** Baseline characteristics of conceived women with PCOS according to FHD.

Characteristic	Overall *N* = 320	Negative FHD *N* = 256	Positive FHD *N* = 64	*p* value
Anthropometric information				
Age, years	27.68 ± 3.21 (320)	27.63 ± 3.20 (256)	27.88 ± 3.29 (64)	0.592
Height, cm	1.61 ± 0.05 (320)	1.61 ± 0.05 (256)	1.62 ± 0.05 (64)	0.192
Weight, kg	61.32 ± 11.57 (320)	60.45 ± 11.73 (256)	64.79 ± 10.31 (64)	0.004
Waist circumference, cm	84.12 ± 11.41 (320)	83.21 ± 11.33 (256)	87.73 ± 11.08 (64)	0.005
Hip circumference, cm	97.91 ± 8.76 (320)	97.23 ± 8.81 (256)	100.62 ± 8.04 (64)	0.004
BMI	23.63 ± 4.06 (320)	23.35 ± 4.13 (256)	24.74 ± 3.55 (64)	0.008
WHR	0.86 ± 0.07 (320)	0.85 ± 0.07 (256)	0.87 ± 0.06 (64)	0.090
WHtR	0.52 ± 0.07 (320)	0.52 ± 0.07 (256)	0.54 ± 0.07 (64)	0.013
Systolic blood pressure, mmHg	111.87 ± 9.65 (320)	111.59 ± 9.87 (256)	112.97 ± 8.71 (64)	0.275
Diastolic blood pressure, mmHg	74.41 ± 7.24 (320)	74.71 ± 7.24 (256)	73.19 ± 7.18 (64)	0.133
Baseline fasting serum testing				
Glucose and insulin metabolism				
Fasting insulin, U/L	88.53 ± 85.80 (305)	89.79 ± 93.23 (244)	83.48 ± 45.45 (61)	0.450
Glucose, mmol/L	5.01 ± 0.91 (309)	4.99 ± 0.95 (246)	5.08 ± 0.75 (63)	0.448
HOMA-IR	2.98 ± 3.70 (302)	3.04 ± 4.06 (241)	2.76 ± 1.63 (61)	0.413
Lipids and lipoproteins				
HDL, mmol/L	1.30 ± 0.40 (309)	1.33 ± 0.40 (246)	1.20 ± 0.36 (63)	0.019
LDL, mmol/L	2.89 ± 0.79 (308)	2.83 ± 0.75 (245)	3.11 ± 0.89 (63)	0.022
Triglyceride, mmol/L	1.47 ± 0.88 (308)	1.41 ± 0.78 (245)	1.71 ± 1.15 (63)	0.049
APOA1, g/L	1.52 ± 0.33 (308)	1.54 ± 0.33 (245)	1.46 ± 0.29 (63)	0.084
APOB, g/L	0.87 ± 0.26 (308)	0.84 ± 0.25 (245)	0.95 ± 0.29 (63)	0.006
Cholesterol, mmol/L	4.65 ± 1.01 (308)	4.59 ± 0.99 (245)	4.87 ± 1.07 (63)	0.063
Lipoprotein, mg/L	124.47 ± 93.77 (308)	118.56 ± 78.20 (245)	147.48 ± 137.10 (63)	0.112
Sexual hormones				
LH, mIU/mL	10.71 ± 6.41 (306)	10.89 ± 6.36 (245)	9.98 ± 6.58 (61)	0.331
FSH, mIU/mL	6.16 ± 1.82 (305)	6.25 ± 1.80 (244)	5.80 ± 1.88 (61)	0.099
Progesterone, nmol/L	2.98 ± 6.48 (305)	3.19 ± 7.18 (245)	2.12 ± 1.61 (60)	0.034
Estradiol, pmol/Lnb	323.08 ± 462.21 (307)	331.31 ± 413.50 (246)	289.90 ± 624.51 (61)	0.624
Total testosterone, nmol/L	1.63 ± 0.65 (307)	1.62 ± 0.65 (245)	1.64 ± 0.64 (62)	0.881
Free testosterone, pg./mL	2.19 ± 0.83 (307)	2.20 ± 0.85 (245)	2.16 ± 0.74 (62)	0.672
SHBG, nmol/L	46.54 ± 31.62 (309)	48.74 ± 33.24 (248)	37.62 ± 21.98 (61)	0.002
LH to FSH ratio	1.85 ± 1.43 (305)	1.81 ± 1.10 (244)	1.99 ± 2.33 (61)	0.551
Free androgen index	5.14 ± 3.94 (306)	5.01 ± 4.00 (245)	5.66 ± 3.70 (61)	0.232
Menstrual features				
Average number of menstruations/years, times	6.52 ± 1.87 (320)	6.57 ± 1.75 (256)	6.33 ± 2.26 (64)	0.418
Menstruation duration, days	63.20 ± 34.86 (320)	61.93 ± 34.86 (256)	68.27 ± 34.65 (64)	0.195
Age at menarche	13.71 ± 1.58 (320)	13.80 ± 1.60 (256)	13.33 ± 1.45 (64)	0.023
Delivery history				
Previous deliveries, no.				0.323
0	303/320 (94.7%)	240/256 (93.8%)	63/64 (98.4%)	
1	16/320 (5.0%)	15/256 (5.9%)	1/64 (1.6%)	
2	1/320 (0.3%)	1/256 (0.4%)	0/64 (0.0%)	
Previous spontaneous abortions, no.				0.084
0	265/320 (82.8%)	218/256 (85.2%)	47/64 (73.4%)	
1	49/320 (15.3%)	34/256 (13.3%)	15/64 (23.4%)	
2	6/320 (1.9%)	4/256 (1.6%)	2/64 (3.1%)	
Duration of attempting pregnancy, months	20.71 ± 15.25 (305)	20.73 ± 15.37 (244)	20.62 ± 14.87 (61)	0.959
Clinical phenotypes				
Metabolic Syndrome	37/308 (12.0%)	27/245 (11.0%)	10/63 (15.9%)	0.401
Hypertension	34/320 (10.6%)	29/256 (11.3%)	5/64 (7.8%)	0.555
PCOM	279/296 (94.3%)	224/239 (93.7%)	55/57 (96.5%)	0.624
HA	212/311 (68.2%)	165/249 (66.3%)	47/62 (75.8%)	0.197
Biochemical HA	181/306 (59.2%)	138/245 (56.3%)	43/61 (70.5%)	0.062
Clinical HA	76/320 (23.8%)	61/256 (23.8%)	15/64 (23.4%)	>0.999
Amenorrhea	27/320 (8.4%)	16/256 (6.3%)	11/64 (17.2%)	0.010
Partner features				
Partner age, years	29.31 ± 3.79 (318)	29.27 ± 3.86 (254)	29.47 ± 3.48 (64)	0.693
Sperm concentration	118.76 ± 162.08 (318)	118.06 ± 168.15 (254)	121.53 ± 136.52 (64)	0.863

AMH, anti-Mullerian hormone; APOA1, apolipoprotein A1; APOB, apolipoprotein B; BMI, body mass index; BP, blood pressure; FHD, family history of diabetes; FSH, follicle-stimulating hormone; HA, Hyperandrogenism; HDL, high density lipoprotein; HOMA-IR, Homeostatic Model Assessment for Insulin Resistance; LDL, low density lipoprotein; LH, luteinizing hormone; SHBG, sex hormone binding globulin. ^a^*p* values were calculated using the Kruskal-Wallis rank sum test, and Pearson chi-square test as appropriate.

The conception rates in the full PCOS cohort were similar between negative FHD group and positive group (31.9% vs. 32.7%, *p* = 0.844; Supplementary Table S1). Likewise, no statistically significant differences were observed in overall clinical pregnancy or live birth rates in the full cohort (Supplementary Table S1). However, when the analysis was restricted to women who conceived, those with a positive FHD had a lower clinical pregnancy rate than those without FHD (57.8% vs. 70.7%, absolute difference 12.9%, *p* = 0.048; Table 2). Live birth was also numerically lower in the FHD-positive group, although the difference did not reach statistical significance (54.7% vs. 66.4%, *p* = 0.081; Table 2). There was no difference observed in obstetric outcomes between the two groups in women with PCOS (Supplementary Table S2).

**Table 2:** Pregnancy outcomes of conceived women with PCOS according to FHD.

Reproductive outcomes	Overall	Negative FHD	Positive FHD	*p* value
Clinical pregnancy	218/320 (68.1%)	181/256 (70.7%)	37/64 (57.8%)	0.048
Live birth	205/320 (64.1%)	170/256 (66.4%)	35/64 (54.7%)	0.081

The results of the association between FHD and clinical pregnancy among conceived women with PCOS are presented in Table 3. In the unadjusted modified Poisson model, positive FHD was associated with a lower probability of clinical pregnancy, although the association did not reach statistical significance (Model 1: RR = 0.82, 95% CI: 0.65–1.02, *p* = 0.078). The direction of association remained similar after adjustment for intervention, preconception reproductive history, partner factors, age, progesterone, SHBG, and anthropometric confounders (Models 2–4). After additional adjustment for lipid parameters (HDL, LDL, triglyceride, and ApoB), the association was further attenuated (Model 5: RR = 0.85, 95% CI: 0.66–1.08, *p* = 0.178), suggesting that these lipid profiles might statistically explain part of the observed association between FHD and clinical pregnancy. The E-values for the point estimates ranged from 1.65 to 1.81, indicating that modest unmeasured confounding could potentially explain the observed associations; therefore, these findings should be interpreted cautiously.

**Table 3:** Stepwise modified Poisson regression models for clinical pregnancy among women who conceived.

Model	*N*	Events	RR^a^ (95% CI)	*p* value	*e*-value
Model 1	320	218	0.82 (0.65, 1.02)	0.078	1.75
Model 2	302	204	0.82 (0.65, 1.03)	0.092	1.73
Model 3	287	195	0.80 (0.63, 1.02)	0.070	1.81
Model 4	287	195	0.81 (0.63, 1.03)	0.088	1.78
Model 5	283	193	0.85 (0.66, 1.08)	0.178	1.65

Model 1: Family history of diabetes. Model 2: Model 1 + intervention ^b^, duration of attempting pregnancy, previous delivery, previous spontaneous abortion, partner age, sperm concentration. Model 3: Model 2 + age, progesterone, SHBG. Model 4: Model 3 + weight, waist circumference, hip circumference. Model 5: Model 4 + HDL, LDL, triglyceride, ApoB. ^a^RR was estimated using modified Poisson regression with robust standard errors. ^b^Adjustment for treatment allocation (four-arm randomized trial group: acupuncture plus clomiphene, sham acupuncture plus clomiphene, acupuncture plus placebo, sham acupuncture plus placebo).

**Figure 2:**
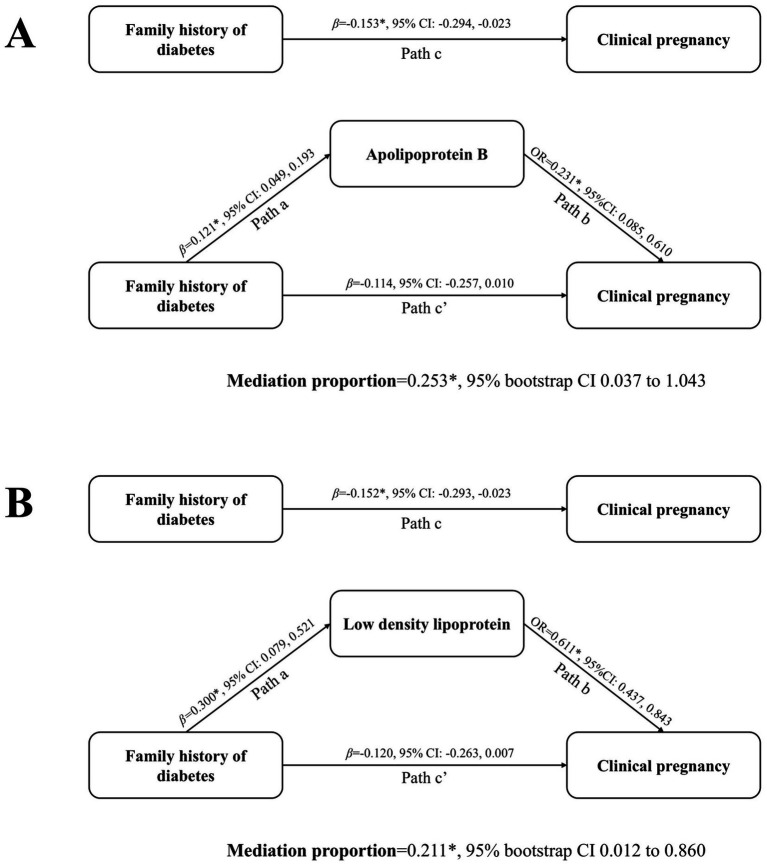
Predictive mediation analysis of (**A**) ApoB and (**B**) LDL in the association between FHD and clinical pregnancy.

Predictive mediation analyses were performed to formally assess whether baseline lipid parameters statistically explained part of the association between FHD and clinical pregnancy (Supplementary Table S3). ApoB and LDL each accounted for a modest proportion (25.3 and 21.1%, respectively) of the observed association between FHD and lower clinical pregnancy in conceived women with PCOS (Figure 2). By contrast, no significant indirect effect was observed for HDL, whereas triglyceride showed a borderline pattern, with a nominally significant indirect effect but a non-significant mediation proportion.

**Figure 3:**
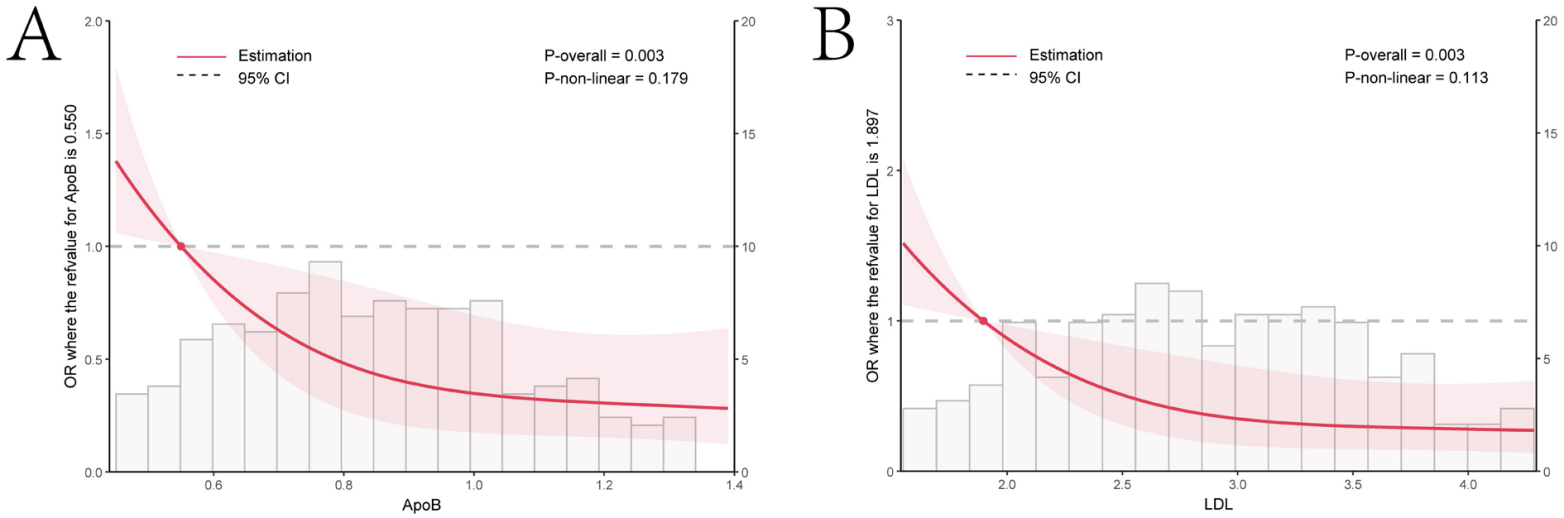
Restricted cubic spline curves for the associations of (**A**) ApoB and (**B**) LDL with clinical pregnancy.

We employed RCS models to evaluate the dose–response relationships between lipid profiles and clinical pregnancy (Figure 3). Both ApoB and LDL exhibited significant overall negative associations with clinical pregnancy (*P*_overall_ = 0.003 for both). The tests for non-linearity were not significant (ApoB: *P*_non-linear_ = 0.179; LDL: *P*_non-linear_ = 0.113), indicating a linear dose-dependent decline. Compared to the reference points (ApoB = 0.550 g/L; LDL = 1.897 mmol/L), the odds of achieving a clinical pregnancy decreased continuously as circulating ApoB and LDL levels increased.

## Discussion

In this secondary analysis, we found that women with positive FHD had a relatively lower clinical pregnancy rate in women with PCOS who conceived, whereas conception rates were similar according to FHD status in the full cohort. ApoB and LDL statistically explained part of this association. In this study, ApoB and LDL are better interpreted as baseline intermediate biomarkers or markers of adverse metabolic risk that statistically explained part of the observed association, rather than as definitively established causal mediators. These findings suggest that the association of FHD with reproductive outcomes may become more apparent after conception rather than at the stage of conception itself.

The primary finding of this research was the negative effect of FHD on clinical pregnancy rates in women with PCOS who conceived. However, direct evidence revealing this association remains limited. Previous studies demonstrated the adverse effects of FHD on reproductive outcomes. In 229 PCOS women, family history of T2DM was found to be the potential risk factor leading to miscarriage (37). Our study showed that FHD + patients had worse clinical pregnancy rates, which matched previous research. However, most previous research focused how FHD affects metabolic features of their daughters, and how metabolism of women affects reproductive outcomes. For example, FHD increased the risk of gestational diabetes (38). Additionally, FHD was associated with placental vascular abnormalities (39), and could cause delayed fetal brain activity (40). Collectively, these findings suggest that FHD adversely affects reproductive success in PCOS patients, with our study identifying reduced clinical pregnancy as a key manifestation.

We also found the positive associations of FHD with increased risk of metabolic disorders in PCOS patients, including higher BMI, WHtR, LDL, triglyceride, ApoB, and lower HDL. This finding suggested that genetic background might play a regulatory role in the development of metabolic features in PCOS. Previous evidence showed that FHD was associated with higher BMI, LDL and triglyceride (15, 41, 42), and much lower HDL (43), which was in accordance with our study. Another research of 2059 participants in young Korean adults indicated that women with positive FHD involving first-degree relatives had higher triglyceride and fasting glucose (44). Parental diabetes might influence the BMI in PCOS, independent of age (45). Previous studies have also indicated that PCOS patients with a FHD tended to have a higher risk of metabolic abnormalities, and this association persisted even after adjusting for confounding factors. Specifically, PCOS patients with a positive FHD often exhibited more significant IR, manifested as elevated levels of HOMA-IR, and fasting serum glucose, while their HbA1c and WHR were also often in a less ideal state (42). Stratified analysis further revealed that patients with a positive FHD may have significantly increased levels of triglyceride, TC, LDL, and HOMA-IR. Among them, those with a first-degree relative with FHD usually faced a higher risk of glucose metabolism disorders compared to those with a second-degree relative with FHD (41).

Based on these observations, the pathogenesis of PCOS was closely related to metabolic factors such as IR, obesity, and dyslipidemia. Obesity and hyperandrogenism could jointly contribute to dyslipidemia (46, 47). Meanwhile, FHD might exacerbate the interplay between IR and hyperandrogenism, promote triglyceride synthesis, aggravate lipid metabolism disorders, and ultimately lead to elevated TC and LDL levels and reduced HDL levels (48, 49). In summary, FHD appears to be an important contributing factor that worsens metabolic risk in PCOS patients. From a nutritional perspective, these fin.

dings are particularly significant because many of these metabolic abnormalities, especially dyslipidemia, are modifiable through dietary interventions. Therefore, for PCOS patients with a positive FHD, early screening of metabolic indicators is recommended, along with the development of personalized intervention strategies that may include targeted nutritional approaches to optimize lipid profiles prior to conception.

A key finding of this study is that higher ApoB and LDL statistically accounted for part of the association between positive FHD and lower clinical pregnancy. Several biologically plausible mechanisms may underlie this association. Successful implantation and early pregnancy maintenance depend on coordinated endometrial receptivity, vascular adaptation, and early placental development (39, 50–52). In women with PCOS, a more atherogenic lipid profile may reflect a metabolic environment associated with oxidative stress, endothelial dysfunction, and impaired microvascular remodeling, which could be less favorable for progression from conception to clinical pregnancy (51–55). Taken together, these observations support the interpretation that ApoB and LDL are clinically informative biomarkers associated with poorer post-conception reproductive outcomes, while mechanistic confirmation requires dedicated prospective studies.

Low-grade inflammation may also be relevant to the observed association. PCOS is increasingly recognized as a condition characterized not only by endocrine and metabolic disturbances but also by chronic low-grade inflammation (56). In this context, atherogenic lipoproteins may represent only one component of a broader lipoinflammatory environment related to impaired reproductive outcomes (39, 55, 56). Dyslipidemia and inflammation may act synergistically, amplifying oxidative stress, endothelial injury, and tissue dysfunction (55–57). In addition, oxidized LDL-C has been implicated in inflammatory activation, apoptosis, and cellular dysfunction through multiple signaling pathways (55), and has also been linked to endothelial dysfunction and inflammation in pregnancy-related complications (58, 59). Because these lipid parameters are potentially modifiable, they may represent clinically relevant targets for future preconception risk stratification and intervention studies.

Our study also revealed that pre-pregnancy triglyceride was negatively associated with clinical pregnancy. However, most previous research focused on the relationships between triglyceride and pregnancy complications, and there is limited research directly on how pre-pregnancy triglyceride affects reproductive outcomes. For example, higher triglyceride in early pregnancy was found to be associated with pregnancy complications and adverse outcomes (60). Additionally, triglyceride can be an indicator of IR in patients with recurrent pregnancy loss, even without PCOS or obesity (61). Other studies also connected higher triglyceride to preterm delivery (62, 63). These findings suggest that elevated triglyceride may also be associated with an unfavorable reproductive environment and poorer clinical pregnancy outcomes.

This study has several strengths. To our knowledge, it is the first to elucidate the potential role of ApoB and LDL as partial metabolic biomarkers linking FHD to impaired clinical pregnancy in PCOS. It effectively bridges the gap between genetic family history and reproductive outcomes by identifying intermediate risk factors. Furthermore, the analysis is based on a large-scale, multicenter randomized controlled trial (PCOSAct), ensuring standardized, high-quality data collection and enhancing the robustness of our findings.

However, several limitations must also be acknowledged. First, this was a secondary analysis of the conceived subgroup within an RCT, and the sample size may have limited statistical power for some exploratory analyses. In addition, because the primary analysis of clinical pregnancy was restricted to women who conceived, conditioning on conception may have introduced selection bias, including possible collider stratification bias. Second, lipid parameters were assessed only at baseline, and we did not have serial measurements across the peri-conception period or early pregnancy. Third, the mediation analysis was based on observational relationships among baseline variables and outcome, so causal interpretation requires caution and residual confounding cannot be excluded (64). Although FHD and lipid parameters were measured at the same baseline visit, FHD reflects an underlying familial predisposition that precedes the measured metabolic phenotype. We therefore treated baseline lipids as potential intermediate biomarkers that may statistically explain part of the association with later clinical pregnancy, rather than as confirmed causal mediators. Fourth, inflammatory and vascular biomarkers were not available in the current dataset; therefore, although inflammation and endothelial dysfunction represent biologically plausible mechanistic pathways, they could not be directly evaluated. Future prospective studies with repeated metabolic measurements and concurrent assessment of inflammatory, endothelial, and endometrial markers are needed to clarify the mechanistic pathway linking FHD to impaired reproductive outcomes in PCOS.

In conclusion, positive FHD was associated with a reduced clinical pregnancy rate among women with PCOS who conceived, whereas no statistically significant association was observed for live birth in the present analysis. Elevated ApoB and LDL partially explained this association, and might serve as markers of an adverse metabolic function related to poorer post-conception reproductive outcomes. These findings highlight the potential value of baseline lipid assessment in women with PCOS and FHD, but they should be interpreted cautiously given the predictive mediation analyses, the restriction to women who conceived, and the possibility of residual confounding. Further prospective and mechanistic studies are needed.

## Data Availability

The data presented in this study are available from the corresponding author upon request with permission from the principal investigators of the original trial.

## Glossary

ApoB: Apolipoprotein B
BMI: Body mass index
CLBR: Cumulative live birth rate
FAI: Free androgen index
FHD: Family history of diabetes
FSH: Follicle-stimulation hormone
GDM: Gestational diabetes mellitus
HA: Hyperandrogenism
hCG: Human Chorionic Gonadotropin
HDL: High-density lipoprotein
HOMA-IR: homeostasis model assessment of insulin resistance
ICSI: Intracytoplasmic Sperm Injection
IR: Insulin resistance
IVF: *In vitro* Fertilization
LDL: Low-density lipoprotein
LFR: LH to FSH ratio
LH: Luteinizing hormone
MS: Metabolic syndrome
RR: Risk ratio
oxLDL: Oxidized LDL-C
PCOM: Polycystic ovary morphology
PCOS: Polycystic ovary syndrome
PCOSAct: Acupuncture and Clomiphene in Polycystic Ovary Syndrome Trial
RCS: Restricted cubic spline
SHBG: Sex hormone binding globulin
T2DM: Type 2 diabetes
TC: Total cholesterol
WHR: Waist to hip ratio
WHtR: Waist to height ratio
