# Supplementary material for: ApoB and LDL partially explain the association between family history of diabetes and lower clinical pregnancy in women who conceived with PCOS
Source: Front Nutr. 2026 May 25;13:1819555. doi: 10.3389/fnut.2026.1819555 (PMC13243417; doi:10.3389/fnut.2026.1819555)
Supplement: Supplementary file 1 [file Table_1.docx]

Table S1. Association between family history of diabetes and reproductive outcomes of PCOS patients in full cohort.

|  | Negative FHD  N=802 | Positive FHD  N=196 | P value |
| --- | --- | --- | --- |
| Ovulation | 616/802 (76.8%) | 163/196 (83.2%) | 0.054 |
| Conception | 256/802 (31.9%) | 64/196 (32.7%) | 0.844 |
| Clinical pregnancy | 181/802 (22.6%) | 37/196 (18.9%) | 0.262 |
| Live birth | 170/802 (21.2%) | 35/196 (17.9%) | 0.299 |
