# Supplementary material for: ApoB and LDL partially explain the association between family history of diabetes and lower clinical pregnancy in women who conceived with PCOS
Source: Front Nutr. 2026 May 25;13:1819555. doi: 10.3389/fnut.2026.1819555 (PMC13243417; doi:10.3389/fnut.2026.1819555)
Supplement: Supplementary file 2 [file Table_2.docx]

Table S2. Association between family history of diabetes and obstetric outcomes in women who conceived.

|  | Negative FHD  N=168 | Positive FHD  N=35 | P value |
| --- | --- | --- | --- |
| Gestational age, days | 274.18 ± 12.53 | 271.83 ± 15.17 | 0.33 |
| Gender |  |  | 0.94 |
| Male | 78/168 (46.4%) | 16/35 (45.7%) |  |
| Female | 90/168 (53.6%) | 19/35 (54.3%) |  |
| Birth weight, g | 3337.46 ± 574.73 | 3327.57 ± 593.33 | 0.93 |
| Body length, cm | 50.17 ± 2.03 | 49.90 ± 1.97 | 0.49 |
| NICU admission rate, n (%) | 15/168 (8.9%) | 3/35 (8.6%) | 0.95 |
| 1 min Apgar score ≥8, n (%) | 125/130 (96.2%) | 30/31 (96.8%) | 0.87 |
| 5 min Apgar score ≥8, n (%) | 123/126 (97.6%) | 29/29 (100%) | 0.40 |
