# Supplementary material for: ApoB and LDL partially explain the association between family history of diabetes and lower clinical pregnancy in women who conceived with PCOS
Source: Front Nutr. 2026 May 25;13:1819555. doi: 10.3389/fnut.2026.1819555 (PMC13243417; doi:10.3389/fnut.2026.1819555)
Supplement: Supplementary file 3 [file Table_3.docx]

Table S3. Predictive bootstrap-based mediation analyses evaluating whether baseline lipid parameters statistically explained part of the association between FHD and clinical pregnancy. Confidence intervals were estimated using 1000 bootstrap resamples.

| Effects | Estimate | 95% CI | *P* value |
| --- | --- | --- | --- |
| FHD→HDL→clinical pregnancy |  |  |  |
| Total effect | -0.154 | -0.294, -0.025 | 0.018 |
| Indirect effect | 0.009 | -0.011, 0.032 | 0.352 |
| Direct effect | -0.163 | -0.309, -0.038 | 0.016 |
| Mediation proportion | -0.059 | -0.467, 0.097 | 0.362 |
| FHD→LDL→clinical pregnancy |  |  |  |
| Total effect | -0.152 | -0.293, -0.023 | 0.018 |
| Indirect effect | -0.032 | -0.067, -0.004 | 0.016 |
| Direct effect | -0.120 | -0.263, 0.007 | 0.062 |
| Mediation proportion | 0.211 | 0.012, 0.860 | 0.034 |
| FHD→ApoB→clinical pregnancy |  |  |  |
| Total effect | -0.153 | -0.294, -0.023 | 0.020 |
| Indirect effect | -0.039 | -0.079, -0.009 | 0.006 |
| Direct effect | -0.114 | -0.257, 0.010 | 0.072 |
| Mediation proportion | 0.253 | 0.037, 1.043 | 0.026 |
| FHD→Triglyceride→clinical pregnancy |  |  |  |
| Total effect | -0.154 | -0.297, -0.024 | 0.020 |
| Indirect effect | -0.025 | -0.065, -0.001 | 0.042 |
| Direct effect | -0.129 | -0.273, -0.006 | 0.040 |
| Mediation proportion | 0.163 | -0.006, 0.666 | 0.062 |
